# Functional brain network centrality is related to APOE genotype in cognitively normal elderly

**DOI:** 10.1002/brb3.1080

**Published:** 2018-08-22

**Authors:** Alle Meije Wink, Betty M. Tijms, Mara ten Kate, Eva Raspor, Jan C. de Munck, Ellemarije Altena, Mirian Ecay‐Torres, Montserrat Clerigue, Ainara Estanga, Maite Garcia‐Sebastian, Andrea Izagirre, Pablo Martinez‐Lage Alvarez, Jorge Villanua, Frederik Barkhof, Ernesto Sanz‐Arigita

**Affiliations:** ^1^ Department of Radiology, Nuclear Medicine and PET Research Neuroscience Campus Amsterdam VU University Medical Centre Amsterdam The Netherlands; ^2^ Department of Neurology Alzheimer Centre Neuroscience Campus Amsterdam VU University Medical Centre Amsterdam The Netherlands; ^3^ Department of Physics and Medical Technology Neuroscience Campus Amsterdam VU University Medical Centre Amsterdam The Netherlands; ^4^ Université Bordeaux Bordeaux France; ^5^ CNRS SANPSY USR 3413 Bordeaux France; ^6^ CITA Alzheimer Foundation Donostia University Hospital San Sebastian Spain; ^7^ Donostia Unit, Osatek Donostia University Hospital San Sebastian Spain; ^8^ Institutes of Neurology and Healthcare Engineering University College London London UK

**Keywords:** Alzheimer's disease, amyloid, APOE‐ε4, eigenvector centrality, functional MRI, visual cortex

## Abstract

**Introduction:**

Amyloid plaque deposition in the brain is an early pathological change in Alzheimer's disease (AD), causing disrupted synaptic connections. Brain network disruptions in AD have been demonstrated with eigenvector centrality (EC), a measure that identifies central regions within networks. Carrying an apolipoprotein (APOE)‐ε4 allele is a genetic risk for AD, associated with increased amyloid deposition. We studied whether APOE‐ε4 carriership is associated with EC disruptions in cognitively normal individuals.

**Methods:**

A total of 261 healthy middle‐aged to older adults (mean age 56.6 years) were divided into high‐risk (APOE‐ε4 carriers) and low‐risk (noncarriers) groups. EC was computed from resting‐state functional MRI data. Clusters of between‐group differences were assessed with a permutation‐based method. Correlations between cluster mean EC with brain volume, CSF biomarkers, and psychological test scores were assessed.

**Results:**

Decreased EC in the visual cortex was associated with APOE‐ε4 carriership, a genetic risk factor for AD. EC differences were correlated with age, CSF amyloid levels, and scores on the trail‐making and 15‐object recognition tests.

**Conclusion:**

Our findings suggest that the APOE‐ε4 genotype affects brain connectivity in regions previously found to be abnormal in AD as a sign of very early disease‐related pathology. These differences were too subtle in healthy elderly to use EC for single‐subject prediction of APOE genotype.

## 1 INTRODUCTION

### 1.1 The role of amyloid‐β in Alzheimer's disease

The accumulation of amyloid‐β (Aβ) plaques in the brain is one of the first events in the pathological cascade leading to Alzheimer's disease (AD) (Bateman et al., 2012; Hardy & Selkoe, 2002; Sperling et al., 2010). Aβ disrupts synaptic functioning, resulting in aberrant brain connectivity at the synaptic level (Selkoe, 2002; Spires‐Jones & Hyman, 2014), as well as on the whole‐brain connectivity level (Delbeuck, der Linden, & Collette, 2003; Hedden et al., 2009). Although the precise sequence of events caused by Aβ plaques is still being investigated (Altmann, Ng, Landau, Jagust, & Greicius, 2015) and interactions with other agents recognized (Jones et al., 2017), the key role of Aβ in AD pathology is beyond doubt (Jack et al., 2016).

### 1.2 The APOE‐ε4 allele and Aβ pathology

Carrying the APOE‐ε4 allele is the main genetic risk factor for Aβ deposition (Ba et al., 2016; Verghese, Castellano, & Holtzman, 2011; Yu, Tan, & Hardy, 2014). Recent findings show that the ε4 isoform of the ApoE protein is less efficient in clearing Aβ compared to ε2 and ε3, leading to accelerated aggregation of plaques. Early imaging studies show hypometabolism in AD‐specific regions in APOE‐ε4 carriers (Reiman et al., 1996). Carriers have also shown detrimental effects on cognition in old age (Deary et al., 2002). Recent results show the correlation of APOE‐ε4 carriership with Aβ deposition, cognition, and brain atrophy (Bonham et al., 2016; ten Kate et al., 2016; Lim & Mormino, 2017). APOE‐ε4 carriers show abnormal Aβ plaque deposition at relatively younger ages (Jansen et al., 2015; Strittmatter, Weisgraber, et al. 1993).

### 1.3 Brain connectivity and resting‐state functional MRI

Brain connectivity is disrupted in AD (Binnewijzend et al., 2012; Tijms et al., 2013, 2014), indicating that AD is a disconnectivity disorder. Resting‐state functional MRI (RS‐fMRI) detects functional connections in the brain as synchronized activity between brain regions in the absence of a task (Binnewijzend et al., 2012). Brain regions linked to AD pathology in studies using MEG and structural MRI (de Haan, Mott, et al., 2012; de Haan, van der Flier, et al., 2012; Tijms et al., 2013) show high connectivity in RS‐fMRI; they are *hub regions*. The high vulnerability of hubs for AD is also found in RS‐fMRI studies (Buckner et al., 2009; Qi et al., 2010). The default mode network (DMN) and other resting‐state networks, that is, regions with synchronized fMRI activity, have been studied as markers for AD progression (Binnewijzend et al., 2012; Filippini et al., 2009; Sheline et al., 2010). Functional brain connectivity changes in healthy adults are related to amyloid depositions (Hedden et al., 2009; Sperling et al., 2009), and carriers of the APOE‐ε4 allele show increased co‐activation with the DMN in young adults (Bookheimer et al., 2000; Filippini et al., 2009), indicating that functional connectivity is sensitive to AD‐related alterations of the brain. This study extends these previous findings, using a large sample from a population imaging study, combined with comprehensive AD‐related cognitive assessment and genotyping.

### 1.4 Functional brain network hubs: eigenvector centrality and its relevance to AD

In graph theory, the notion of centrality (Bavelas, 1948) expresses the amount of network traffic going through a network node. Eigenvector centrality (EC) can be efficiently computed from whole‐brain connectivity matrices derived from RS‐fMRI or electroencephalographic (EEG) data (Lohmann et al., 2010). Eigenvector centrality is sensitive to changes in brain connectivity on different levels of the network hierarchy (Binnewijzend et al., 2014). Previous studies suggest that EC is used as a proxy marker for mild cognitive impairment (MCI) (Meinzer et al., 2012) and AD (Binnewijzend et al., 2014), where patients have decreased EC compared to healthy controls in occipital regions. Because of its sensitivity to AD‐related changes and its low computational cost, EC is a useful measure for generating biomarkers for AD pathology from RS‐fMRI data.

Although it is not currently known at which point in the development of the disease these EC changes occur, it has shown sensitivity to changes before the stage of cognitive decline and irreversible brain atrophy. This stage of milder cognitive problems, accompanied by localized changes in brain metabolism and functioning (Jack et al., 2010, 2013), provides an opportunity for treatment (Cummings & Zhong, 2014; Hampel et al., 2010). Biomarkers related to disease progression in these early stages are essential for proper quantitative evaluation. If EC detects AD‐related network alterations in the presymptomatic stage of the disease or in people with a well‐defined risk for AD, this will further increase opportunities for treatment development (Cummings & Zhong, 2014; Jack et al., 2013).

The aim of this study was to determine whether eigenvector centrality mapping (ECM) can detect early changes related to genetic risk of AD in cognitively normal adults. In a specific manner, we expected that healthy subjects at increased risk for AD due to the APOE‐ε4 genotype would show locally decreased EC compared to noncarriers, and this would correlate with nonimaging markers and tests used for AD. At last, we explored the predictive value of ECM for AD risk.

## 2 METHODS AND MATERIALS

### 2.1 Participants

Participants were recruited via the media between 2011 and 2013 as part of the networks@risk project at CITA‐Alzheimer, San Sebastian, Spain, for the Gipuzkoa Alzheimer Project (GAP), a longitudinal population study of AD risk in the Basque region of Spain (Martinez‐Lage et al., 2013), approved by the Gipuzkoa clinical research ethics committee. The sampled population was a group of healthy community‐dwelling participants aged 39–80 years old. Healthy subjects without memory complaints, with a clinical dementia rating (CDR) < 1 and a Mini‐Mental State Examination (MMSE) test score of at least 28, were included. Exclusion criteria were any psychiatric, neurological, or systemic symptoms that could cause cognitive deficits, resulting in a representative sample of the healthy citizens in this age group.

Subjects gave written informed consent; the study was approved by the Gipuzkoa medical and research ethical committee. Visits included MR scanning, medical tests and interviews, as well as extensive neuropsychological testing. The Framingham cardiovascular risk index, which is strongly correlated with the probability of dementia and AD (D'Agostino et al., 2008), was computed for each participant to be used as a covariate for removing cardiovascular risk confounds.

### 2.2 APOE genotyping

APOE genotype was obtained using one‐stage PCR as previously described (Alcolea et al., 2014; Martinez‐Lage et al., 2013) and dichotomized as no *APOE‐ε*4 allele (*APOE*4−) or at least one *APOE‐ε*4 allele (*APOE*4+). Risk for AD was defined by APOE genotype, with APOE4+ being the high‐risk group (Strittmatter, Saunders, et al. 1993) as in previous studies of these data (ten Kate et al., 2016; Tijms et al., 2016). Further subdivisions by genotype were not possible because of the low number of ε2 carriers (*N* = 19) and ε4 homozygotes (*N *= 5).

### 2.3 MR acquisition

Structural imaging included T1‐weighted MRI on a 3T scanner (Tim Trio, Siemens, Erlangen, Germany) using a magnetization‐prepared rapid gradient echo (MPRAGE) sequence, 1.25‐mm isotropic resolution. Functional imaging included resting‐state functional MRI (RS‐fMRI) while the subjects were lying still with their eyes closed, trying to stay awake, and not to focus on anything specific with an echo‐planar imaging (EPI) sequence, 325 volumes, a repetition time (TR) of 1.82 s, an echo time (TE) of 30 ms flip angle of 90°, a 3.3 mm slice thickness, and 3.0 × 3.0 mm pixels. To reduce scanning time, the EPI slice stack had partial brain coverage and was oriented to include the regions of the DMN to be detected in a separate analysis. Normalized gray matter volume (NGMV) as a fraction of total brain volume was computed using the segmented MPRAGE scans with the IBA‐SPM toolbox (http://www.thomaskoenig.ch/Lester/ibaspm.htm).

### 2.4 Image preprocessing

All DICOM images were converted to NIfTI using MRIcron (Rorden, Karnath, & Bonilha, 2007). The structural scans were stripped of nonbrain tissue using the VBM8 toolbox (see http://dbm.neuro.uni-jena.de/vbm). The rest of the processing was performed using FSL (Smith et al., 2004) as follows. The structural images were mapped into the standard MNI space (Mazziotta et al., 2001) using a spline‐based nonlinear registration algorithm (Rueckert et al., 1999) implemented in FSL as FNIRT. The volumes in the RS‐fMRI data were stripped of nonbrain tissue (Smith, 2002) and spatially realigned to the middle volume of the time series using FSL MC‐FLIRT (Jenkinson, Bannister, Brady, & Smith, 2002). This program measures relative mean voxel displacement, which is very similar to mean frame displacement FD (Power, Barnes, Snyder, Schlaggar, & Petersen, 2012). The realigned data were spatially smoothed with a 3D Gaussian filter (full width at half the maximum 3.3 mm isotropic), and an edge‐preserving nonlinear filter (Smith & Brady, 1997). Time series were high‐pass filtered at a cutoff frequency of 182 s (100 TR). The RS‐fMRI data of each subject were mapped to the native‐space structural MR image using boundary‐based registration (Greve & Fischl, 2009), after which the standard space‐mapping parameters of the structural image were used to map them to MNI standard space at a sampling resolution of 4 mm isotropic. Of the initially selected study of 269 subjects with fMRI data, eight had to be discarded due to bad image quality (excessive motion, e.g., too high FD values as evaluated and reported by the preprocessing software (mean displacement > 0.5 mm), missing data and/or failed registration to the anatomical scans), leaving 261 preprocessed fMRI data sets. Two separate versions of the preprocessed data were used: one that was preprocessed as above, and one where the effects of motion were computed in single‐subject GLMs with the realignment parameters as covariates, and then subtracted from the data.

### 2.5 Eigenvector centrality mapping

Eigenvector centrality mapping (ECM) of the standard‐space RS‐fMRI data was performed using fast ECM (Wink, de Munck, van der Werf, van den Heuvel, & Barkhof, 2012), a memory‐ and time‐efficient implementation of ECM using the connectivity matrix *R *+* *1, where *R* is the voxelwise correlation matrix. This measure is the relative difference of two normalized time signals on a positive scale from 0 to 2. With non‐negative connectivities, the Perron–Frobenius theorem guarantees positive values in the dominant eigenvector (Wink et al., 2012). The fastECM algorithm allows the computation of this eigenvector without the need to compute or store *R* explicitly, thus increasing efficiency and enabling fast computations at high resolutions (see https://github.com/amwink/bias/tree/master/matlab/fastECM). Centrality was only computed inside the intersection of all single‐subject masks based on the standard‐space fMRI data to ensure the network topology under investigation did not differ between subjects, simplifying between‐group comparisons (van Wijk, Stam, & Daffertshofer, 2010). Single‐subject masks were made by computing the temporal minimum for each 4D volume. Two separate ECM were computed for each subject: one with, and one without the motion parameters regressed out as explained before.

### 2.6 Statistical analysis

#### 2.6.1 Eigenvector centrality differences between risk groups

Maps of voxelwise group mean EC values were computed for APOE4+ and 4− groups separately. Significant differences between the APOE4+ and APOE4− groups were computed in a group‐level general linear model (GLM) whose design included gender, age, NGMV, and Framingham index as covariates. Significance was computed based on permutation testing of group labels, using cluster mass as a test statistic (Bullmore et al., 1999). A cluster‐forming threshold was automatically determined to maximize the number of suprathreshold clusters in the null distribution. Cluster mass statistics were computed in the observed and null data; the cluster mass threshold for significance was set to yield at most one expected false‐positive cluster per image. The group analysis was performed twice: once for the original ECM, and once for the ECM with the motion parameters regressed out. To exclude effects from correlations introduced by motion or conversely, by the regression of motion parameters, the cluster for subsequent testing was computed as the intersection of the results of these two analyses, that is, voxels that were found in both tests, and the values of the ECM after motion regression were used.

#### 2.6.2 Relation of eigenvector centrality and markers of AD risk

Mean centralities were computed for every subject inside the cluster mask. Correlations of EC values with biological and neuropsychological markers of AD risk were computed with R (http://www.r-project.org, version 3.3.3). Linear fits were plotted of cluster mean ECM against age and scores on the psychological tests and CSF biomarker levels, grouped by genetic risk (APOE4− vs. APOE4+). Separate one‐way analyses of variance (ANOVA) with group as the factor determined the effect of the markers and group on the mean EC. Differences in mean EC were assessed by ANOVA of group mean +individual means, for both groupings separately.

#### 2.6.3 Use of cluster mean ECM as a predictor for AD risk

To test the usability of single‐subject cluster mean EC as a predictor of genetic AD risk, a logistic regression was used to predict the APOE risk of each subject, using cluster mean EC as the predictor and age as a covariate. The regression was computed in the GLMnet package for R (Friedman, Hastie, & Tibshirani, 2010) with the model without elastic net penalization, that is, using the ordinary least squares solution. The model was evaluated with leave‐one‐out cross‐validation. A receiver operating curve (ROC) of the model was constructed using the pROC package for R (Robin et al., 2011); 95% confidence intervals were computed using bootstrap resampling. This procedure was repeated for a second model with age as a second regressor.

## 3 RESULTS

### 3.1 Study characteristics

Of the total sample, 76 (29%) individuals were APOE‐ε4 carrier subjects (Table 1). MMSE scores were not statistically different between APOE4+ risk groups (Kruskal–Wallis *χ* ^2^ = 1.51, *p* = 0.22). Scores on the 15‐object test (15OT) were higher for the APOE4+ risk group (median APOE4− 13; median APOE4 + 14; Kruskal–Wallis test *p* = 0.025). No significant risk group‐related differences were found for gender (Kruskal–Wallis *χ* ^2^ = 7.1 × 10^−5^, *p* = 0.99), age (Kruskal–Wallis *χ* ^2^ = 3.41, *p* = 0.065), normalized gray matter volume (NGMV, Kruskal–Wallis *χ* ^2^ = 0.92, *p* = 0.761), or cardiovascular risk (Kruskal–Wallis *χ* ^2^ = 0.194, *p* = 0.660) between the high and low genetic risk groups (see Table 1).

**Table 1:** Group characteristics of the high‐risk (APOE‐ε4 carriers) and low‐risk groups in the sample

	Whole sample	APOE4− group	APOE4+ group	*p*
Number of subjects	261	185 (71%)	76 (29%)	
Male/female	110/151	78/107	32/44	0.993
Age mean/*SD*	56.6/6.7	57.0/6.8	55.6/6.6	0.065
MMSE mean/*SD*	29.1/0.8	29.0/0.7	29.1/0.8	0.219
15OT score mean/*SD*	13.1/1.78	13.0/1.86	13.5/1.51	0.025*
Framingham CV risk	6.46/6.17	6.41/6.16	6.61/6.27	0.660
NGMV mean/*SD*	0.452/0.019	0.452/0.019	0.453/0.020	0.761

Note

The groups did not differ significantly for gender and age distributions (*p* = 0.993 and *p* = 0.065, respectively). The APOE4+ groups did not differ in mean MMSE scores (*p* = 0.219). The APOE4+ risk group scored higher on the 15‐object test (15OT, *p* = 0.025). The groups did not differ significantly in cardiovascular risk scores and NGMV (*p* = 0.660 and *p* = 0.761, respectively). Significant differences are marked with *.

### 3.2 Eigenvector centrality differences between risk groups

**Figure 1:**
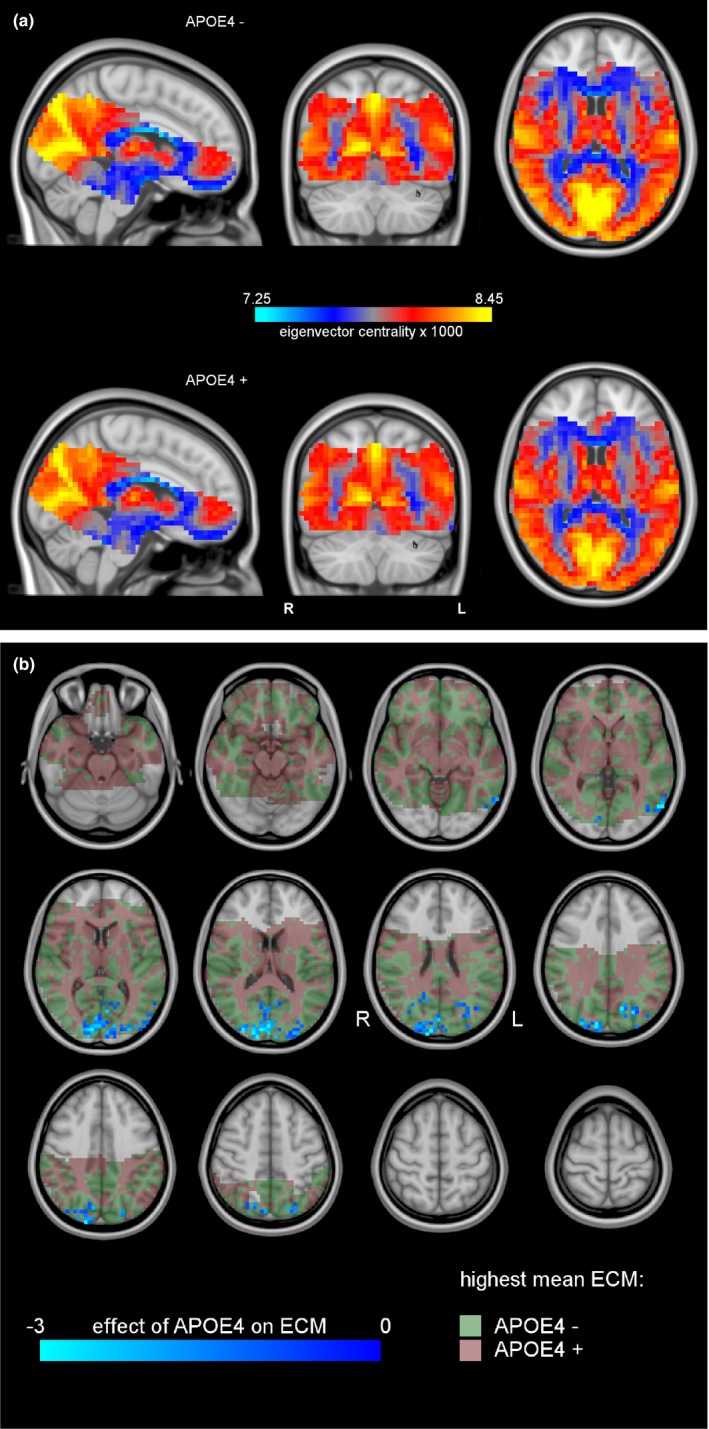
(a) Group mean ECM for the APOE4− group (top) and the APOE4+ group (bottom). Centralities could only be computed in brain regions that were scanned in every subject (colored part). Blue indicates relatively low centralities, and yellow indicates high centralities. The occipital region indicated by the green arrow has visibly higher voxelwise centralities in the low‐risk group than in the high‐risk group. (b) Cluster of significant ECM differences between the APOE4− and APOE4+ groups (blue). The anatomical background is shown in gray scales. Areas where the mean centrality is highest in the APOE4+ group are in red, and areas where the mean centrality was highest in the APOE4− group are in green

Figure 1a shows the average EC for the APOE4− and APOE4+ groups, respectively. The APOE+ group average showed areas of decreased EC compared to APOE−, mostly in the occipital areas. Figure 1b shows the cluster of significantly decreased EC in the APOE4+ group: bilaterally in the occipital pole (V1 and V2, Brodmann areas 17 and 18), extending into the left and right superior lateral occipital lobes, dorsal posterior cingulate cortex (PCC, Brodmann area 31), and the precuneus, with a total size of 387 voxels (24,768 mm³). The areas in green and red shades show (a) the mask in which the EC was computed (green + red) and (b) the regions with higher EC in the APOE4+ risk group (red) and lower (in green). Most of the cortical regions show a lower mean centrality for the APOE4+ group. (Xia, Wang, & He, 2013).

### 3.3 Correlations of EC with nonimaging markers

**Figure 2:**
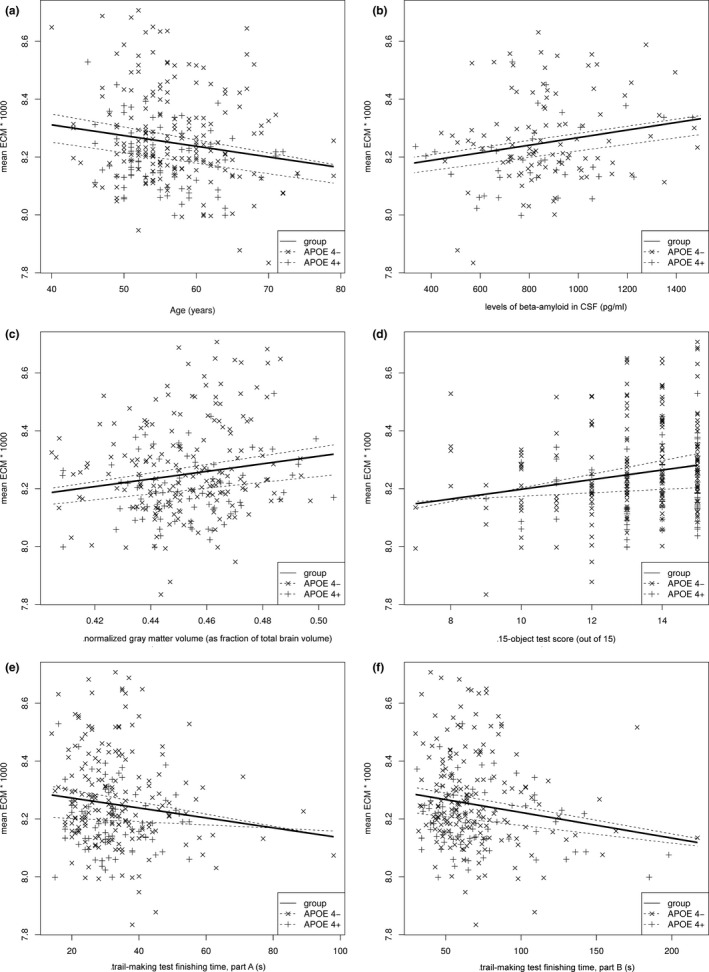
Cluster mean EC of all subjects plotted against subject age (a), CSF amyloid levels (b), NGMV (c), 15‐object test score (15OT) (d), and time to complete the trail‐making test (TMT) part A (e) and part B (f)

For each subject, we extracted the mean EC inside the occipital cluster (Figure 1b) to explore associations with the other risk factors. Regression of these means against age, the main risk factor for AD, showed a significant negative effect of age (*p* ≤ 0.007, see Table 2). Separate fits of the APOE subgroups independently showed that this effect (Figure 2a) was statistically significant in the low‐risk APOE4− subgroup, but not in the APOE4+ subgroup (*p* = 0.012 vs. *p* = 0.058) and there were no significant interactions. Cluster mean EC was significantly negatively correlated with CSF amyloid levels (*p* = 0.018, see Figure 2b). *p*‐Values for the APOE4− and APOE4+ subgroups were 0.085 and 0.159, respectively. There was a significantly negative correlation with NGMV (*p* = 0.008) with *p*‐values in the APOE4− and APOE4+ subgroups of 0.022 and 0.113, respectively (Figure 2c).

**Table 2:** Correlations of biological markers of AD risk with EC means measured inside the cluster of significant between‐group differences (see Figure 1b)

Effect of interest	*p* value	APOE 4−/4+	Linear fits in subgroups	
			APOE4−	APOE4+
Age	*p* = 0.007*	*p* = 5.0 × 10^−5^ *	*p* = 0.012* *R* ^2^ = 0.034	*p* = 0.058 *R* ^2^ = 0.048
CSF‐amyloid	*p* = 0.017*	*p* = 0.037*	*p* = 0.085 *R* ^2^ = 0.030	*p* = 0.159 *R* ^2^ = 0.056
NGMV	*p* = 0.008*	*p* = 1.4 × 10^−4^ *	*p* = 0.022* *R* ^2^ = 0.028	*p* = 0.113 *R* ^2^ = 0.034
15OT test score	*p* = 0.001*	*p* = 1.6 × 10^−5^ *	*p* = 0.000* *R* ^2^ = 0.071	*p* = 0.481 *R* ^2^ = 0.007
Time for TMT pt. A	*p* = 0.030*	*p* = 1.4 × 10^−4^ *	*p* = 0.032* *R* ^2^ = 0.025	*p* = 0.665 *R* ^2^ = 0.003
Time for TMT pt. B	*p* = 0.005*	*p* = 3.1 × 10^−4^ *	*p* = 0.035* *R* ^2^ = 0.024	*p* = 0.102 *R* ^2^ = 0.036

Note

The * indicates *p* <0.05

There were three statistically significant correlations of cluster mean EC with cognitive markers. First, the 15OT (*p* = 0.001 for the whole group; *p* < 0.001 and *p* = 0.481 for the APOE4− and APOE4+ subgroups, respectively; see Figure 2d) and also the time required for the trail‐making test, parts A (*p* = 0.030) and B (*p* = 0.006). For both parts of the test, the correlations are stronger in the APOE4− subgroup than APOE4+ (part A: *p* = 0.032 vs. *p* = 0.665, part B: *p* = 0.035 vs. *p* = 0.102; see Figure 2e,f).

### 3.4 Use of cluster mean ECM as a predictor for AD risk

**Figure 3:**
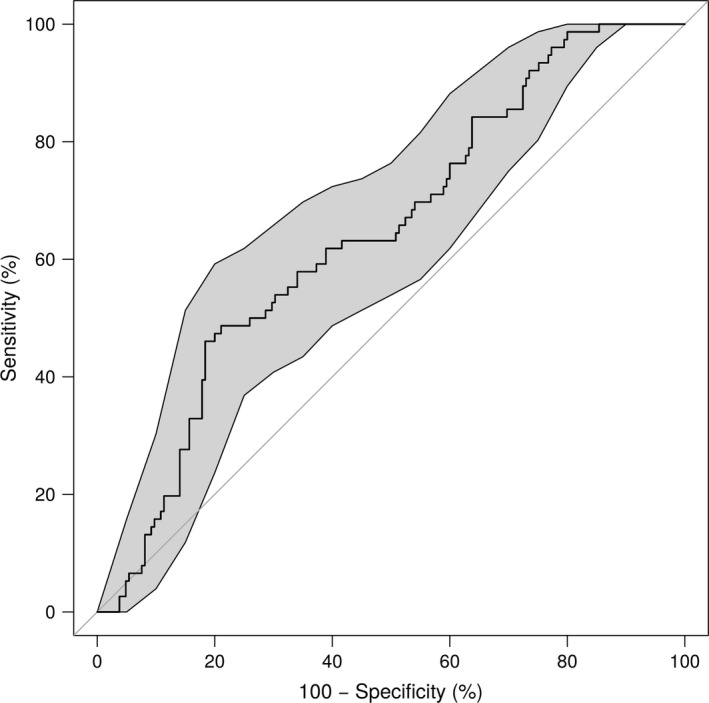
Receiver operating curve (ROC) for predicting the APOE risk group with a logistic regression, using the mean ECM in the cluster of between‐group differences (see Figure 2b) as the main predictor and age and 15‐object test scores as covariates. The shaded areas are the 95% confidence intervals. The area under the curve (AUC) measured with this model was 64.4%

The cross‐validation of the logistic regression produced an accuracy of 68.9%, and the ROC corresponding to the parameter λ with the lowest validation error showed an area under the curve (AUC) of 64.4% (see Figure 3). Adding age to the model did not improve the results (accuracy was 68.6%; AUC was 64.5%).

## 4 DISCUSSION

The main result of this study is that APOE‐ε4 carriers show decreased EC in comparison with noncarriers in visual cortical regions; which have previously been demonstrated to be affected in MCI and AD (Rombouts et al., 2009; Sanz‐Arigita et al., 2010). Furthermore, lower EC values were associated with older age and worse visual task performance in noncarriers.

### 4.1 APOE‐ε4‐related differences in functional brain eigenvector centrality

Our findings show that functional whole‐brain network centrality changes may already be present in cognitively normal older adults who have an increased risk of developing AD. Previous studies that have measured functional brain changes with fMRI between AD patients, MCI patients and healthy controls (Binnewijzend et al., 2012; Drzezga et al., 2011; Ossenkoppele et al., 2013), have demonstrated functional changes in early stages of AD. Our findings are in line with these previous observations, showing that these functional changes are also present in carriers of the APOE‐ε4 allele, a risk factor for AD.

The regions of changed centrality partially coincide with previous work that reported brain regions with default mode network (DMN)‐related changes in APOE‐ε4 carriers (Filippini et al., 2009), most markedly the precuneus. Those did not include the visual cortex as the statistical analyses were limited to changes inside the DMN. Analyses of connectivity with the rest of the brain (Agosta et al., 2012) show differential connectivity with the DMN with the same visual regions we report, between AD and controls as well as MCI vs. controls, especially on the boundary between precuneus and visual cortex.

Findings of connectivity changes inside and to the DMN in relation to APOE‐ε4 carriership have not been consistent, mostly due to the different age groups being studied (Filippini et al., 2011; Heise, Filippini, Ebmeier, & Mackay, 2011; Mevel, Chételat, Eustache, & Desgranges, 2011). Overall, the most frequently reported changes are weakened DMN connectivity in middle‐aged and older subjects (Goveas et al., 2013; Machulda et al., 2011; Reiman et al., 1996; Sheline et al., 2010; Wang et al., 2012) and, less frequently, increased DMN connectivity in young adults (Filippini et al., 2009; Fleisher et al., 2009). Our results demonstrate early visual effects in a population study with realistic proportions of carriers and noncarriers, which shows the applicability of functional measures in a community setting.

Recent studies that focus less on the DMN alone report a shift of functionally central and highly connected regions from posterior to frontal regions in AD patients (Agosta et al., 2012; Binnewijzend et al., 2014; Sanz‐Arigita et al., 2010; Sheline et al., 2010). The decreased centralities of posterior regions in the APOE4+ risk group found in this study are in line with this shift, although we did not find locally increased centralities in frontal areas in healthy subjects.

Functional brain network changes related to (risk of) AD have mainly been studied using techniques that detect the DMN. Tested as a whole, patients exhibit lower DMN connectivity than controls, see (Wang et al., 2015; Lee et al., 2016) and their references. The “canonical” pattern of the DMN is the precuneus, superior lateral parietal lobes, and the ACC. When analyses are limited to the DMN regions, this is where the changes are found (Binnewijzend et al., 2012; Filippini et al., 2009). Analyses of AD‐related functional brain network differences associated with the DMN but not part of the DMN report changes in superior parietal and occipital regions (Agosta et al., 2012; Lee et al., 2016). These findings are consistent with those in resting and visual fMRI studies that report affected visual functioning accompanied by differences in the visual cortices (Alegret et al., 2010; Lehmann et al., 2013; Wang et al., 2015; Zhang et al., 2010) and the idea that deviations from the typical pathology, such as the involvement of functionally specific brain regions, drive the variation in neurodegenerative variation in AD.

The decreased EC values, we have found in the visual cortex in the APOE risk group, show strong similarities to the posterior regions of decreased centralities in advanced AD patients (Binnewijzend et al., 2014), indicating that the onset of centrality changes measured in advanced AD are detectable in healthy patients with an elevated risk for AD. If both sets of regions disclose the same process, a possible explanation is that the aberrant functional connectivity in the brain associated with the APOE‐ε4 allele makes it more vulnerable to AD‐related pathology.

This is in line with earlier findings of a change in “hub” status of these brain regions in AD patients (Buckner et al., 2009; de Haan, Mott, et al., 2012; de Haan, van der Flier, et al., 2012), and AD‐related changes in glucose metabolism (Ossenkoppele et al., 2013) and neuronal activity (Damoiseaux et al., 2012; Navas et al., 2013).

The GAP cohort of healthy elderly with documented AD risk fills the gap of the less frequently studied group of elderly, healthy adults with known APOE‐ε4 genotype, and our study confirms the persistence of these genotype‐driven changes from young adults and elderly healthy stages to early and advanced stages of AD. The efficiency of fast ECM (Wink et al., 2012) and its sensitivity to disease conditions (Binnewijzend et al., 2014), and the fact that it produces single‐subject, whole‐brain patterns, make it an attractive alternative to current RS‐fMRI analyses of AD‐related brain network differences, for example, independent component analyses. As such, whole‐brain network analyses are an interesting and novel approach to multiple‐network decompositions for neurological disorders that perturb the global brain network functionally and structurally (Agosta et al., 2012; Seeley, Crawford, Zhou, Miller, & Greicius, 2009; Tijms et al., 2014).

### 4.2 Relation between occipital EC and nonimaging biomarkers

Older subjects showed lower eigenvector centrality values and a significant negative correlation with age across groups. (Tijms et al., 2014; van der Flier, Pijnenburg, Fox, & Scheltens, 2011) This decrease is in line with recent results from fMRI studies in healthy middle‐aged controls, where different levels of activity in V1 discriminate between controls with risk of AD who carry the APOE‐ε4 allele from those who do not (Rajah et al., 2017).

We found a positive correlation with CSF amyloid levels, which corresponds to a negative correlation with amyloid load in the brain. This is consistent with the finding that ECM correlates negatively with age, because brain amyloid load correlates positively with age (Oh, Madison, Baker, Rabinovici, & Jagust, 2016).

Cluster mean EC was also related to a decrease in normalized gray matter volume (NGMV). Atrophy differences in this cohort related to APOE genotype have been reported previously but were limited to the striatum and insula (ten Kate et al., 2016).

Correlations between EC and scores on the 15‐object test (15OT) and trail‐making test (TMT) were all positive. For the TMT, times to finish did not differ significantly between the groups. For the 15OT, there was also a difference in scores between the groups: The APOE4+ group had higher scores. This finding is somewhat counterintuitive, as the APOE4+ group scored higher on the 15OT. The plots per subgroup show that EC variability against test score is lower in e4 carriers. This indicates differences in brain network organization between carriers and noncarriers that make the brain more vulnerable to AD‐related pathology (De Meyer et al., 2010; Evans et al., 2014; Mintun et al., 2006; Sperling et al., 2011). In an important way, this does not lead to decreased cognitive decline or visuoperceptual performance. Indeed, previous studies have reported a positive effect of APOE‐ε4 on cognition in young and middle‐aged adults, suggesting that APOE‐ε4‐related changes are beneficial in early life but detrimental in old age (Bunce, Anstey, Burns, Christensen, & Easteal, 2011; Rusted et al., 2013). Improved attention in young APOE‐ε4 carriers is one of the fundamental cognitive differences recently reported (Rusted et al., 2013).

When stratified for APOE4 genotype, we found that correlations of EC with other markers were significant only for the APOE4− group. Cluster mean EC variability is lower within the APOE4+ high‐risk group. The mean EC is significantly higher in the low‐risk group than in the high‐ risk group, so the absence of the age effect in the latter could point to a localized decrease in centrality earlier in life for the high‐risk subjects (Tijms et al., 2014; van der Flier et al., 2011). Another possible explanation is selection bias by removing subjects with low MMSE. If only subjects with APOE and/or amyloid are included, they may have a higher “cognitive reserve”: They may be able to perform better with (more) brain pathology (van Loenhoud et al., 2017).

### 4.3 APOE‐ε4‐related centrality changes in the visual cortex

The regions where decreased centrality was detected in this study are in the visual cortex. The visual cortex and regions of the ventral visual processing stream involved in object recognition have been recognized as areas affected by AD in studies of cortical atrophy and using object recognition fMRI tasks (Jacobs et al., 2015). Given the decrease in EC in the primary visual cortex is correlated with lower performance on the 15‐object task, visual perception deficits may be explained by APOE‐related changes, such as posterior atrophy that is specific to the carrier group (Adaszewski, Dukart, Kherif, Frackowiak, & Draganski, 2013; Yao, Hu, Liang, Zhao, & Jackson, 2012).

Our results are partly concordant with recent findings that APOE‐ε4 carriers show different brain activity during scene perception (Shine, Hodgetts, Postans, Lawrence, & Graham, 2015), and anatomically match previously reported cases of AD‐related visuoperceptual deficits (Chan et al., 2015) and studies of posterior cortical atrophy (Crutch et al., 2012; Migliaccio et al., 2012).

### 4.4 Functional brain network centrality as a predictor of APOE genotype

The results for predicting the genetic risk group using the cluster mean EC yielded AUC and accuracy higher than chance, but below 75% (1 in every 4 misclassified). In our sample of healthy elderly, the changes in EC that can be measured between groups are too subtle for single‐subject classification. With a stronger contrast between patients and controls, the discriminating power of regional EC differences may be useful for diagnostic purposes. Although APOE status is a risk factor for AD (Caselli et al., 2009; Ossenkoppele et al., 2013; Strittmatter, Saunders, et al., 1993), many other processes are involved in causing dementia, so the difference between APOE‐ε4 carriers and noncarriers may be a relatively small AD‐related effect on the brain network.

## 5 LIMITATIONS AND FUTURE DIRECTIONS

The limited brain coverage of the slice stack used for the fMRI acquisition does not fully employ the benefits of a whole‐brain network analysis like ECM. The acquisition parameters were chosen to limit scanning time while still capturing the regions of the default mode network (Raichle & Snyder, 2007), a group of regions whose activity and connectivity measures change significantly in patients with AD. Future fMRI studies of brain network changes in AD should provide whole‐brain coverage to make optimal use of modern analysis methods. Another limitation is that it is unknown who will develop AD pathology; this will be clearer in follow‐up studies of this sample that are ongoing.

## 6 CONCLUSION

Using ECM of resting‐state fMRI data in healthy controls, we have identified functional brain network changes in carriers of the APOE‐ε4 allele, a genetic risk factor for AD, which are directly linked to age and cognitive performance in healthy aging.

## CONFLICT OF INTERESTS

No conflict of interests were declared.
